# Typhoidal *Salmonella* Trends in Thailand

**DOI:** 10.4269/ajtmh.18-0046

**Published:** 2018-07-25

**Authors:** Chonnamet Techasaensiri, Amruta Radhakrishnan, Daina Als, Usa Thisyakorn

**Affiliations:** 1 Department of Pediatrics, Faculty of Medicine, Ramathibodi Hospital, Mahidol University, Bangkok, Thailand;; 2 Centre for Global Child Health, The Hospital for Sick Children, Toronto, Canada;; 3 Department of Pediatrics, Chulalongkorn University, Bangkok, Thailand

## Abstract

Typhoid and paratyphoid fever remain endemic diseases in Thailand with wide variation in subnational incidence trends. We examined these trends alongside contextual factors to study potential interactions and guide control strategies for this disease. Culture-confirmed typhoid and paratyphoid fever data from 2003 to 2014 were collected from the Ministry of Public Health website. Contextual factor data were collected from various sources including World Health Organization/United Nations Children’s Fund Joint Monitoring Program, United Education Statistical World Bank database, World Bank, Development Research group, and global child mortality estimates published in the Lancet. Typhoid fever exhibited a declining trend with peak incidence reported in 2003 at 8.6 cases per 100,000 persons per year. Incidence dropped to three cases per 100,000 persons in 2014. The trend in paratyphoid fever remained stable with the peak incidence of 0.77 cases per 100,000 persons observed in 2009. Subnational variations of typhoid were seen throughout the study period with the highest incidence observed in the northwestern region of Thailand. Increases in female literacy, and access to improved water and sanitation were observed with decreases in poverty head count ratio and diarrheal mortality rate per 1,000 live births. Case fatality remained consistently low at 0.4% or less in all years with reported deaths. At the national level, typhoid fever incidence has shown a notable decline; however, incidence appears to have plateaued since 2007 with access to improved water supply and sanitation above 80%. Eliminating this disease will require strong disease prevention measures in conjunction with effective treatment interventions.

## INTRODUCTION

Typhoid and paratyphoid fever, collectively known as enteric fever, are endemic diseases in Thailand with considerable regional variation. In 2013, subnational incidence estimates ranged from 0.9 to 83.6/100,000, highlighting the disparities between provinces.^1^ Following mass vaccination campaigns in the late 1970s, typhoid fever declined considerably in certain areas and national rates of typhoid fever have remained lower than 10/100,000 people in recent years. Paratyphoid fever rates have consistently remained significantly lower than those of typhoid; however, large outbreaks still occur, making enteric fever a persistent public health concern.^1^ Data from the 1970s show that typhoid fever rates steadily increased in highly populated areas such as Bangkok and reached epidemic levels in 1976.^2^ Antimicrobial-resistant strains of *Salmonella* Typhi and *Salmonella* Paratyphi, the causative agents of typhoid and paratyphoid fever, respectively, have been an emerging issue worldwide since the early 1980s.^3^ This led to the commencement of large-scale targeted vaccination programs aimed at immunizing schoolchildren in high-burden areas because they make up the most susceptible age group. A national school-based immunization program began in 1977 targeting children aged 7–12 years.^2^ Over the course of 6 years, children within the program received annual heat- or phenol-inactivated typhoid vaccinations.^2^ The estimated typhoid vaccine coverage during that period was 80% of the eligible population. Following these campaigns, there was a considerable decline in typhoid fever cases in Bangkok; however, a concurrent slight increase in the cases of paratyphoid fever was reported.^2^

Typhoid fever can spread through various pathways, including fecal-oral transmission and ingestion of contaminated water or food. One way in which this disease has been managed in Thailand was through the preventive measure of vaccination. Following the introduction of the national vaccination program, typhoid fever incidence showed a decrease, but it is difficult to determine to what degree the intervention affected typhoid incidence as no official control was in place. In addition to vaccinations, water, sanitation and hygiene (WASH) programs have been implemented to improve water quality and sanitation practices in the country. In 1960, “The Village Health and Sanitation Project” was initiated.^4^ This program partnered with World Health Organization (WHO), UNICEF, and United States Agency for International Development to decrease the prevalence of waterborne diseases by providing training to health workers with the goal of empowering communities to be more engaged in sanitation facility management. The Thai government issued a legislative act to legally create the Metropolitan Waterworks Authority in 1967 and the Provincial Waterworks Authority in 1979 to explore the raw water sources, produce good quality of water supply, and distribute to Bangkok and all other provinces.^5^ The “Health for all by the year 2000” initiative began in 1978. This intervention provided funding to certain villages, known as health development villages, to improve sanitation conditions.^4^ From 1981 to 1990, the intervention in place was known as the “International Drinking Water Supply and Sanitation Decade.” The goal of this intervention was to increase the coverage of water and sanitation from 75% to 90% across the country. The main aim of the present study is to examine the trends in typhoid and paratyphoid fever in Thailand over the past 20 years within the context of relevant contextual factors.

## METHODS

### Data on typhoidal *Salmonella*.

Data on culture-confirmed estimates of typhoid and paratyphoid fever were obtained from the Ministry of Public Health website spanning 2003–2014. The data were stratified by province as well as age group. The national typhoid fever surveillance program in Thailand involves passive reporting of enteric fever cases in conjunction with general disease surveillance.^6^ The surveillance system separately reports estimates for enteric fever, and typhoid and paratyphoid fever. All reported cases of enteric fever here are clinically suspected with positive Widal test, whereas all cases of typhoid and paratyphoid fever reported are culture-confirmed by blood, urine, or stool. We only included culture-confirmed estimates of typhoid and paratyphoid fever for this analysis.

### Data on contextual factors.

A number of factors have been linked to the spread of enteric fever. Data on these factors were collected from various sources. Water, sanitation and hygiene data were collected from the World Bank database and the WHO/UNICEF Joint Monitoring Program. Female literacy data were extracted from the World Bank and United Nations Education Scientific and Cultural Organization. Socioeconomic status was extracted in the form of percentage of the population living on less than 1.90$/day from the Development Research group database of the World Bank. Diarrheal mortality in children younger than 5 years was obtained from a review by Liu et al.^7^ on cause-specific mortality in children younger than 5 years. All factors were extracted at the national level.

### Statistical methods.

Detailed statistical analyses were not undertaken for this study. Data are graphically presented to clearly outline the observed longitudinal trends. Simple linear regression was performed between each contextual factor and typhoid and paratyphoid fever incidence.

## RESULTS

### National incidence trend.

**Figure 1.**
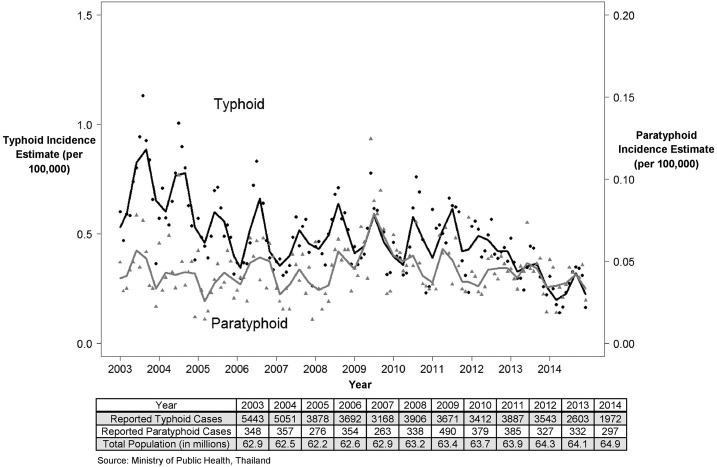
Longitudinal trend in typhoid and paratyphoid fever. The black line depicts the locally weighted scatterplot smoothing curve for national monthly trend in typhoid fever. The black circles represent the monthly incidence per 100,000 persons with typhoid fever in Thailand. The gray line depicts the LOWESS curve for the national monthly trends in paratyphoid fever. The gray triangles represent the monthly incidence per 100,000 persons with paratyphoid fever in Thailand.

At the national level, both typhoid and paratyphoid fever rates in Thailand have shown an overall decreasing trend throughout the duration of the study. The peak incidence of typhoid fever was 8.6 cases per 100,000 persons per year in 2003 (Figure 1). A steady decline was observed until 2007 where incidence was reported at five cases per 100,000 persons per year. In 2008, typhoid incidence increased from the previous year to 6.2 cases per 100,000 persons per year and has since decreased to three cases per 100,000 persons in 2014. Paratyphoid fever has shown a steadier trend than that of typhoid fever. The peak incidence of 0.77 cases per 100,000 persons per year occurred in 2009. The lowest incidence of 0.41 cases per 100,000 persons was seen in 2007.

**Figure 2.**
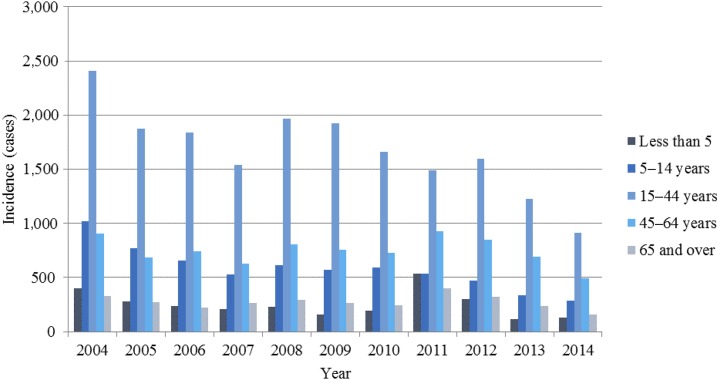
Typhoid fever cases by age group from 2004–2014. The dark blue bars show the proportion of typhoid fever cases in children younger than 5 years. The navy blue bars represent the number of typhoid fever cases in children aged 5–14 years. The pale blue bars indicate the cases of typhoid fever in individuals aged 15–44 years. The sky blue bars show typhoid fever cases in adults aged 45–64 years. The gray–blue bars represent typhoid fever cases in those aged 65 years and older.

The burden of typhoid fever varies by age groups.^8^ In this study, the highest incidence of typhoid was in the age group of 15–44 years, with the peak incidence of 3.8 cases per 100,000 persons per year occurring in 2004 (Figure 2). The lowest reported incidence in this age group occurred in 2014 (1.4 cases per 100,000 persons per year). The lowest typhoid incidence in this study occurred in the age group 65 years and older. The peak incidence in this group was 0.6 cases per 100,000 persons per year in 2011. Typhoid incidence decreased to its lowest point of 0.24 cases per 100,000 persons per year in 2014. Paratyphoid exhibited similar age distribution of incidence, although the number of cases and incidence were considerably lower than that of typhoid. The highest incidence was observed in the age group of 15–44 years and the lowest incidence in the study was again in the age group of 65 years and over.

The typhoid and paratyphoid fever case fatality rates (CFRs) represent the number of deaths caused by typhoid and paratyphoid respectively out of total confirmed cases. Of the patients with confirmed typhoid, fatalities were observed in only 3 years during the study: 2003, 2004, and 2010 during which the CFRs were 0.06%, 0.02%, and 0.03%, respectively. Paratyphoid CFR was calculated in 2014 to be 0.34%. No deaths from paratyphoid were recorded in any other year during the study.

### Subnational incidence trends.

**Figure 3.**
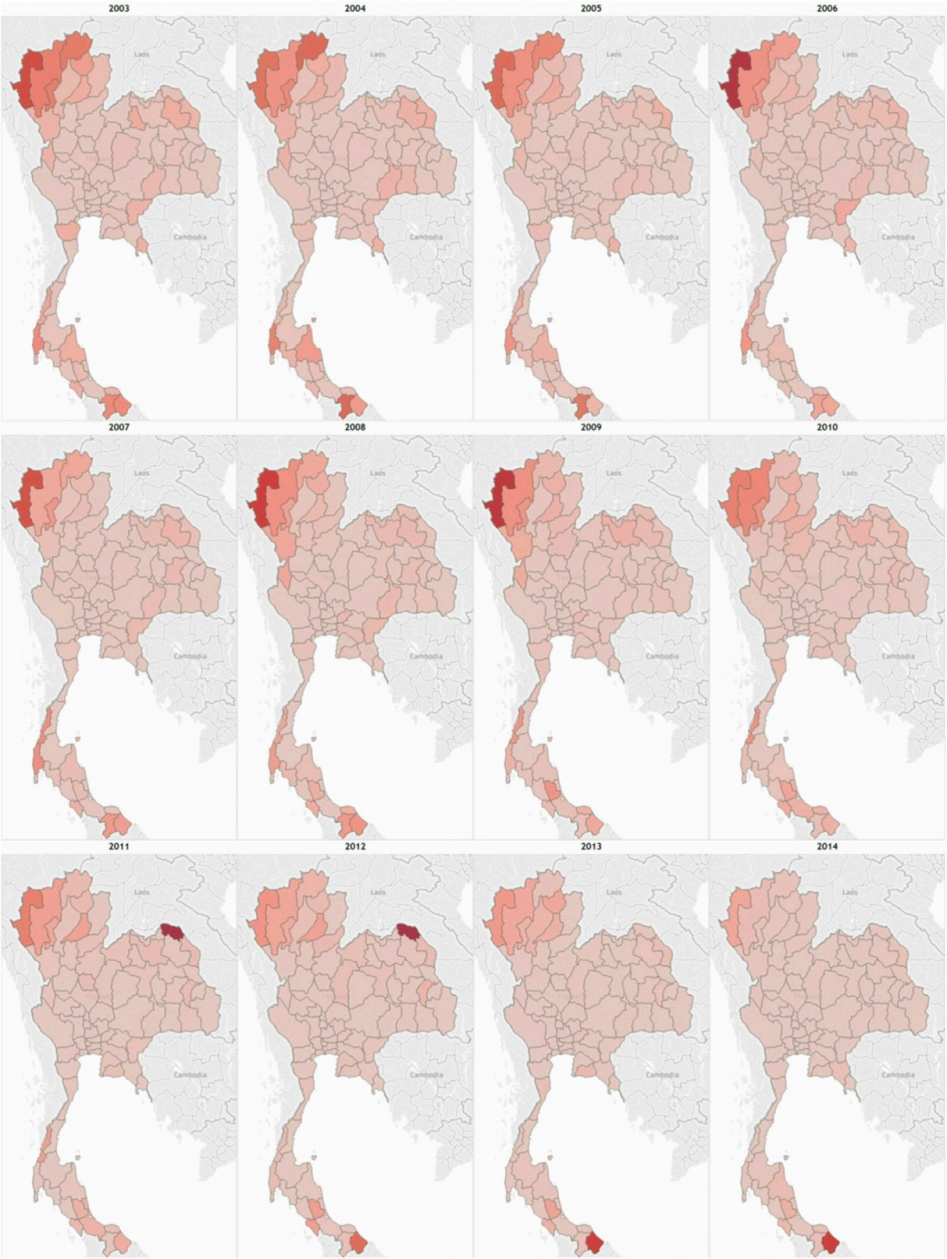
Subnational trends in typhoid fever. Yearly incidence by province, which ranges from 0 to 174.6 per 100,000 persons per year with a single outlier of 686/100,000 persons per year in Bueng Kan, 2011. The darker red shows provinces with high typhoid fever incidence, whereas the provinces with lighter red shading have lower incidence rates per 100,000 persons per year.

Thailand is divided into 76 provinces and Bangkok which is designated as a special administrative area. Figure 3 shows the change in incidence by province from 2003 to 2014. During this time period, national typhoid incidence displayed a generally decreasing trend with the incidence rate per 100,000 people remaining below 10. However, a great degree of variation exists between provinces. The highest burden of typhoid occured in the northwestern region of the country within provinces such as Mae Hong Son, Chiang Mai, Chiang Rai, and Lamphun among others. For most years, incidences in the latter three provinces remained between 20 and 55 per 100,000 people. The province of Mae Hong Son reported the highest provincial incidence rates for the most years with the peak of 158 per 100,000 people in 2006. Although this region as a whole still has the highest incidence of typhoid, declines have been observed over time. Centrally located provinces, including Bangkok and its vicinities have also shown clear decreases in annual typhoid incidence with rates remaining below 2 per 100,000 since 2010. Southern provinces on the other hand display more variation and no clear trend. This variation could be attributed to outbreaks such as the one reported in the province of Songkhla in 2010.

The northeastern provinces of the country generally reported low levels of typhoid fever. In 2011, the new province of Bueng Kan was formed after splitting off from Nong Khai. Following this split, the annual incidence rate dropped to less than 1 per 100,000 in comparison to the earlier rates around 6. The reported incidence rate in the newly formed province of Bueng Kan, however, was 686 cases per 100,000 which is the highest reported provincial incidence rate during the entire study time period. The incidence dropped to 174 in 2012 and 4 in 2013, indicating that there might have been a large-scale epidemic of typhoid in the first year. Rates of paratyphoid fever have remained generally low throughout the country with most provinces reporting zero cases annually. Certain provinces such as Bangkok, Si Sa Ket, and Chiang Rai reported the most cases of paratyphoid, although no clear incidence trend is evident.

Yearly incidence by province ranges from 0 to 174.6 per 100,000 persons per year with a single outlier of 686/100,000 persons per year in Bueng Kan, 2011.

### Patterns of antimicrobial resistance.

**Figure 4.**
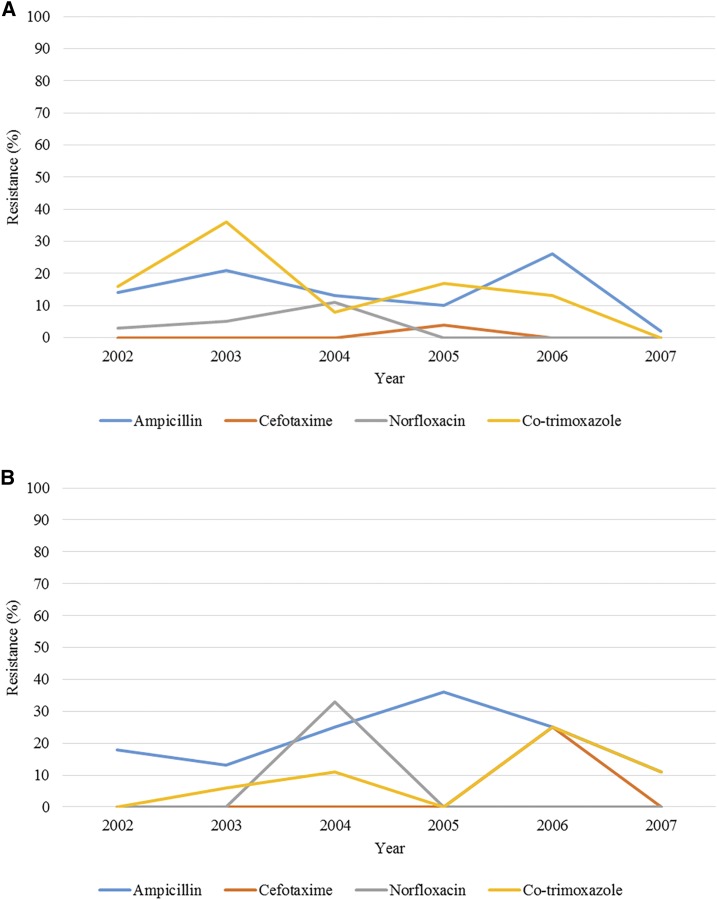
(**A**) *Salmonella* Typhi antimicrobial resistance between 1998 and 2007. The blue line shows the trend in ampicillin-resistant *S.* Typhi isolates. The orange line represents the trend of cefotaxime-resistant *S.* Typhi isolates. The gray line shows the trend of norfloxacin-resistant *S.* Typhi isolates. The yellow line depicts the trend of co-trimoxazole-resistant *S.* Typhi isolates. (**B**) *Salmonella* Paratyphi antimicrobial resistance between 1998 and 2007. The blue line shows the trend in ampicillin-resistant *S.* Paratyphi isolates. The orange line represents the trend of cefotaxime-resistant *S.* Paratyphi isolates. The gray line shows the trend of norfloxacin-resistant *S.* Paratyphi isolates. The yellow line depicts the trend of co-trimoxazole-resistant *S.* Paratyphi isolates.

Although it has been reported that the incidence of antimicrobial-resistant strains of enteric fever is high in Asia,^9^ data specific to Thailand from 1998 through 2007 showed that most of the *S.* Typhi and *S.* Paratyphi were susceptible to cefotaxime and norfloxacin. Antimicrobial susceptibility of *S.* Typhi and *S.* Paratyphi to ampicillin and trimethoprim/sulfamethoxazole were inconsistent.^10^ Across the study period, resistance to the four antibiotics tested (ampicillin, cefotaxime, norfloxacin, and co-trimoxazole) remained below 40% in both *S.* Typhi and *S.* Paratyphi isolates and do not appear to be increasing as of 2007. Figure 4 depicts the resistance of *S.* Typhi and *S.* Paratyphi strains to commonly used antimicrobials over time.

### Trends in contextual factors.

**Figure 5.**
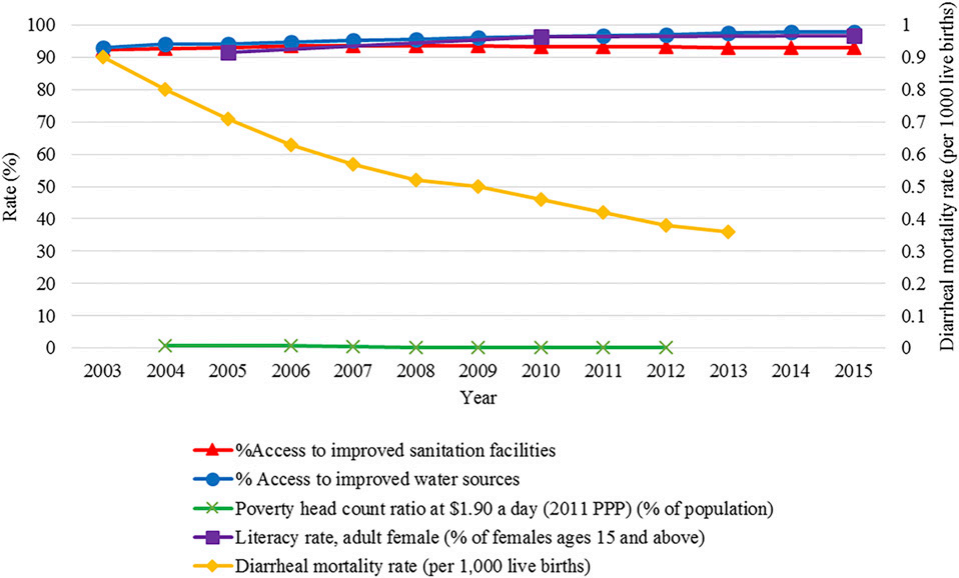
National trends in pertinent contextual factors. A collection of typhoid relevant contextual factor trends are shown from 2003 to 2015. The orange line shows the percentage of the country with access to improved sanitation facilities. The blue line represents the percentage of the population with access to improved water sources. The green line shows the percentage of the population living on less than $1.90 per day. The purple line shows the percentage of literate females aged 15 years and older. The yellow line captured on the secondary *x* axis shows the diarrheal mortality rate per 1,000 live births at the national level.

Contextual factor data were extracted from 2003 to 2014 to align with the available enteric fever data in Thailand (Figure 5). Water, sanitation and hygiene practices are considered to be key factors in enteric fever incidence as the disease can spread through ingestion of contaminated water. Access to improved sanitation facilities as defined by the World Bank is the percentage of the population with flush/pour latrines connected to a sewer, pit or septic tank, ventilated or slab latrines, and composting toilets.^11^ Access to these forms of facilities has plateaued in Thailand at around 93% as of 2006. The types of water sources used for drinking, handwashing, cleaning, and food preparation is important to consider in the management of enteric fever, given the nature of its transmission. The World Bank defines improved water sources as piped water into homes, public taps, standpipes, protected wells, and springs as well as rainwater. The percentage of the population with access to improved water sources has increased slightly across the time period from 93% in 2003 to 98% in 2014.^12^ Socioeconomic status can also contribute to the spread of enteric fever. In this study, we used the World Bank measure of poverty defined as the percentage of the population living on less than 1.90$/day.^13^ This has shown a decrease from 0.75% in 2004 to 0.06% in 2012.^13^ Female literacy data were collected from women aged 15 years and older.^14^ The percentage of females who are able to read showed minimal increase from 91.5% in 2005 to 96.4% in 2010.^14^ Diarrheal mortality, which is linked to a number of waterborne diseases is associated with typhoid. Diarrheal mortality in children younger than 5 years is defined by the WHO as the number of children younger than 5 years dying from diarrhea per 1,000 live births.^15^ The peak for diarrheal mortality during the study period was 1.28 per 1,000 live births in 2000, with a decrease to 0.36 per 1,000 live births in 2013.

A simple linear regression was carried out to determine the relationship between improvements in each contextual factor and the observed incidence trend (Table 1). Based on this analysis, the contextual factors of improved water and decreasing diarrheal mortality in children younger than 5 years were the only factors correlated with decreasing typhoid fever incidence.

**Table 1:** Simple linear regression of contextual factors and typhoid and paratyphoid fever

Contextual factor	Organism	*R* intercept	*R* slope	*R* R2	*R* confidence interval	*P* value
Improved water	*S.* Typhi	81.2	−0.787	0.694	(−1.16, −0.42)	0.0008
Improved sanitation		21.0	−0.163	0.00236	(−2.52, 2.19)	0.8809
Diarrheal mortality		2.48	6.33	0.735	(3.47, 9.19)	0.00074
Poverty rate		5.39	2.01	0.403	(−0.432, 4.447)	0.0908
Improved water	*S.* Paratyphi	0.461	0.000872	0.000227	(−0.0399, 0.0416)	0.9629
Improved sanitation		5.38	−0.0519	0.0640	(−0.1917, 0.08796)	0.4276
Diarrheal mortality		0.592	−0.0687	0.0168	(−0.465, 0.327)	0.7042
Poverty rate		0.581	−0.0340	0.00980	(−0.375, 0.307)	0.8156

## DISCUSSION

Over the study period of 2003–2014, the incidence in typhoid appears to be exhibiting a decreasing trend at the national level. Paratyphoid fever’s trend remained constant and did not demonstrate notable changes in incidence. As contextual factors seem to have remained consistently high across the study period, it is difficult to assess the effect they may have had on the incidence of enteric fever. Although we cannot specifically identify the underlying causes for the observed variation in incidence across age groups, school-aged children have historically been considered to be at a high risk for infection. The data from Thailand aligns with that in the literature depicting the highest burden of typhoid fever in schoolchildren and adolescents.^8^ The targeted vaccination campaigns seem to have contributed to the observed decrease in typhoid fever, most notably in Bangkok where programmatic immunization against typhoid was carried out in the 1970s in school-aged children. If vaccines have in fact been effective at reducing typhoid incidence, the development of a paratyphoid vaccine may be the next step in controlling the incidence of *S*. Paratyphi A, B, and C. A recent study that conducted whole-genome sequencing analysis of typhoidal *Salmonella* isolates noted that most indigenous strains of *S.* Typhi were depleted following the mass vaccination campaigns of the 1970s.^16^ This analysis also indicated that recent strains of *S.* Typhi are closely related to the strains isolated from neighboring countries such as Laos and Cambodia, which implies that the transmission of indigenous strains is not responsible for causing typhoid in Thailand.^16^ Typhoid incidence can potentially be further curbed by using the robust surveillance system in place to improve traveler and migrant screening and, therefore, reduce imported cases of typhoid fever from neighboring endemic countries.

Typhoid and paratyphoid fever are diseases kept under extensive surveillance in Thailand, and this in-depth monitoring likely also contributes to its effective control as outbreaks can be quickly contained. Because typhoid is a notifiable disease, clinicians might be more likely to order blood cultures for febrile patients, leading to quicker diagnoses and treatment commencement in patients.^17^

On account of the comprehensive disease notification system, enteric fever incidence, outbreaks, and mortality rates have been well documented at both the national and subnational level. The distribution of cases by age group and month are also available, which provides insight into the seasonality and age-related burden of the disease. Both typhoid and paratyphoid fever cases peak during the rainy season. Despite the presence of various control measures, disease transmission during these months is likely to increase because the wet season is well suited for typhoidal *Salmonella* bacteria to persist in the environment.^18^

Although most provinces in the country display a decreasing incidence trend, certain provinces in the northwest such as Mae Hong Son, Chiang Mai, Lamphun, and Chiang Rai consistently report some of the highest annual incidences. There are multiple factors likely to be exerting their influence on the observed trends with one potential explanation being the water sources being used in these areas. Most of the northern provinces are rural where communities use a variety of water sources, such as rain water, natural springs, rivers and wells, depending on their needs. As a result, despite the availability of improved water sources, people might use poor and potentially infected sources of water for cooking and cleaning.^19^ In such cases, improved contextual factors at the national level are unlikely to provide an appropriate representation of what is occurring subnationally. The term improved water sources is defined as the delivery of improved water to a population but does not account for the quality of these delivery methods. A region may have access to piped water, but if those pipes are rusted, the quality of the water is no longer safe. Another factor to consider is that the availability of improved water sources does not account for personal practices, and uptake of these resources cannot be captured solely by the measure of access to improved water and sanitation facilities. To gain a better picture of how improved resources are being used, further insight into uptake and household hygiene practices are necessary.

### Limitations.

Although the results presented here illustrate overall disease trends, the absence of reporting on conditions such as typhoid ileal perforations mean that disease severity cannot be ascertained. However, given the low reported CFR throughout the time period, it can be assumed that in most cases, patients made complete recoveries. In addition, contextual factor data were only available at the national level and could not be closely correlated with the observed subnational trends, given the high degree of variability in socioeconomic factors between and within provinces. Although the national disease notification system has considerable benefits, the passive reporting system can lead to underestimating morbidity and mortality. Reporting estimates for enteric fever, and typhoid and paratyphoid fever separately, and based on different test methods, imply that the true burden of disease might be higher than what is reported here. However, given that blood cultures have a higher degree of sensitivity and specificity than Widal tests, the estimates and trends reported here are the most reliable when the national gold standard of diagnosis is being implemented.

Although paratyphoid incidence exhibited a decreasing trend at the national level, certain provinces such as Bangkok and Chiang Rai showed increasing rates over some years in conjunction with decreasing typhoid incidence. Similar inverse trends between typhoid and paratyphoid fever have been observed in certain regions of India, Pakistan, and China as well. Thus, more research into the characteristics of paratyphoid fever and control measures is required.

## CONCLUSION

Nationally, both typhoid and paratyphoid fever exhibited generally decreasing incidence trends since 2003 despite some minor fluctuations. The typhoid and paratyphoid incidence rates do not climb above 9 per 100,000 people and 0.8 per 100,000, respectively. National data regarding relevant contextual factors for the same time period depicted improving trends with access to improved water, sanitation, gross domestic product, and literacy rate increasing, whereas diarrheal mortality and poverty rates decreased. However, despite generally low national rates, considerable regional variation still exists. Longitudinal data from Thailand’s numerous provinces have shown that large-scale outbreaks still occur periodically. In certain areas, paratyphoid incidence has increased, whereas typhoid incidence has decreased. These data suggest that there are many subnational nuances in enteric fever rates that need to be described in more detail before they can be adequately tackled. On a national level, enteric fever rates show a general sign of plateauing between 3 and 8 cases per 100,000 per year. Given that the coverage of WASH infrastructure and other positive contextual factors are above 90% nationally, further curbing the spread of enteric fever by focusing on these areas might not be the most effective option. Context-specific considerations must be taken into account and more granular data regarding the provision and utilization of WASH infrastructure and health promotion campaigns at the subnational level will help with identifying gaps in delivery and implementation. Furthermore, leveraging existing interventions for diarrheal disease control in addition to vaccination campaigns in vulnerable populations in the regions of the country that report the highest enteric fever rates might help mitigate enteric fever morbidity and severity and might also help limit the number of outbreaks.
